# Trajectory of primordial follicle depletion is accelerated in obese mice in response to 7,12-dimethylbenz[a]anthracene exposure^†^

**DOI:** 10.1093/biolre/ioae059

**Published:** 2024-04-16

**Authors:** Jaspreet K Rishi, Kelsey Timme, Hunter E White, Karl C Kerns, Aileen F Keating

**Affiliations:** Department of Animal Science, Iowa State University, Ames, IA 50011, United States; Department of Animal Science, Iowa State University, Ames, IA 50011, United States; Department of Animal Science, Iowa State University, Ames, IA 50011, United States; Department of Animal Science, Iowa State University, Ames, IA 50011, United States; Department of Animal Science, Iowa State University, Ames, IA 50011, United States

**Keywords:** ovary, obesity, DMBA, follicle loss, oxidative stress

## Abstract

Both obesity and exposure to environmental genotoxicants, such as 7,12-dimethylbenz[a]anthracene, negatively impair female reproductive health. Hyperphagic lean KK.Cg-a/a (*n* = 8) and obese KK.Cg-Ay/J (*n* = 10) mice were exposed to corn oil as vehicle control (CT) or 7,12-dimethylbenz[a]anthracene (1 mg/kg/day) for 7d intraperitoneally, followed by a recovery period. Obesity increased liver and spleen weight (*P* < 0.05), and 7,12-dimethylbenz[a]anthracene exposure decreased uterine weight (*P* < 0.05) in obese mice. Primordial follicle loss (*P* < 0.05) caused by 7,12-dimethylbenz[a]anthracene exposure was observed in obese mice only. Primary (lean *P* < 0.1; obese *P* < 0.05) and secondary (lean *P* < 0.05, obese *P* < 0.1) follicle loss initiated by 7,12-dimethylbenz[a]anthracene exposure continued across recovery. Reduced pre-antral follicle number in lean mice (*P* < 0.05), regardless of 7,12-dimethylbenz[a]anthracene exposure, was evident with no effect on antral follicles or corpora lutea number. Immunofluorescence staining of DNA damage marker, γH2AX, did not indicate ongoing DNA damage but TRP53 abundance was decreased in follicles (*P* < 0.05) of 7,12-dimethylbenz[a]anthracene-exposed obese mice. In contrast, increased (*P* < 0.05) superoxide dismutase was observed in the corpora lutea of 7,12-dimethylbenz[a]anthracene-exposed obese mice and reduced (*P* < 0.05) TRP53 abundance was noted in preantral and antral follicles of 7,12-dimethylbenz[a]anthracene-exposed obese mice. This study indicates that obesity influences ovotoxicity caused by a genotoxicant, potentially involving accelerated primordial follicle activation and hampering normal follicular dynamics.

## Teaser

The continuation of ovotoxicity post-cessation of 7,12-dimethylbenz[a]anthracene exposure occurs differentially in lean and obese mice indicating that obesity influences ovotoxicity caused by genotoxicant exposure.

## Graphical Abstract

**Graphical Abstract:**
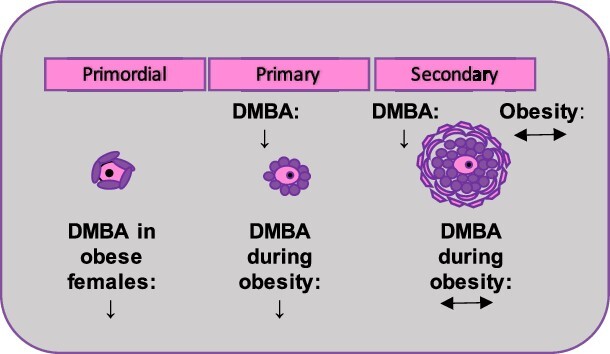

## Introduction

The female gonad, the ovary, is vital for fertility, endocrine homoeostasis, and overall reproductive health. Along with the production, maintenance and ovulation of the female gamete, the ovary also produces the steroid hormones 17β-estradiol (E_2_) and progesterone (P_4_) [1]. Ovarian function can be perturbed by endocrine disruption [2], medical conditions [3], aging [4], environmental toxicant exposure [5], and lifestyle factors including obesity [6].

Under- and over-nutrition both detrimentally affect female reproductive health [7] and obesity is a complex issue caused by poor diet, sedentary lifestyle, lack of physical activity, socio-economic factors, and genetic factors [8]. The dramatic increase in the incidence of obesity in the United States has raised it to epidemic levels [9], and the obesity trend worsened during the COVID-19 pandemic [10]. Among a myriad of other complexities, obesity causes aberration to female reproductive function and is associated with increased anovulation and infertility [11], reduced fecundity [12], endocrine perturbations [13], gestational diabetes [14], pregnancy complications [15], and reduced fertility treatment success [15, 16]. Obesity can alter ovarian function [17, 18] causing basal ovarian DNA damage [19], inducing ovarian inflammation and altering steroidogenic pathways [20]. An altered ovarian response to environmental toxicants is also observed [19, 21–25], suggesting heightened sensitivity to ovotoxicity during obesity.

The polycyclic aromatic hydrocarbon (PAH), 7,12-dimethylbenz[a]anthracene (DMBA), is a model genotoxic and ovotoxic PAH that causes follicle loss in mice and rats [26, 27]. Human exposure to PAH chemicals occurs through smoke from sources including, but not limited to, cigarettes, car exhaust, overheated cooking oil, forest fires, and burning of coal [28]. DMBA is bioactivated in a variety of tissues including the ovary into a genotoxic metabolite, DMBA 3,4-diol, 1,2-epoxide, which forms DNA adducts [29] compromising the integrity and viability of the germ cell.

Genotoxicity induced by DMBA is mainly due to direct DNA binding or generation of reactive oxygen species [29–31] and the ovary responds by initiating the DNA damage response (DDR) pathway. In mice, increased levels of γH2AX are observed after exposure to DMBA [32, 33], indicating the presence of double-stranded DNA breaks. Several ovarian DDR-related proteins, including ATM, PARP1, XRCC1, BRCA1, and RAD51, are also altered by DMBA exposure [22, 32, 33]. In addition, there is an apparent differential DDR in the ovaries of lean and obese mice [19, 21, 25, 33], suggesting that the capacity for DNA repair is compromised in ovaries from obese females, and the DMBA-induced increase in the DDR protein, BRCA1, was impaired by obesity in a follicle-specific manner [22].

A question remains whether immediate effects of exposure to a genotoxicant such as DMBA ceases immediately post-exposure or are those effects ongoing and sustained? In order to address this uncertainty, follicle loss in ovaries from mice who were provided a recovery period post-exposure to DMBA, was compared with follicle loss in ovaries that were collected immediately post-exposure [22]. This permitted testing the hypothesis that post-exposure follicle loss continues in both lean and obese mice. In addition, whether there are markers associated with DDR and follicle viability that remain altered or activated post-exposure was also assessed.

## Materials and methods

### Reagents

Tween was purchased from Fisher Bioreagents (Fair Lawn, NJ, USA). 7,12-dimethylbenz(a)anthracene (CAS # 57-97-6), eosin Y, hematoxylin, paraffin, phosphate-buffered saline (PBS), sodium chloride, and Tris base were from Sigma-Aldrich (St. Louis, MO, USA). Citrasolv, SlowFade Gold mounting media, and SOD1 (PA5 27240) antibody were obtained from Thermo Fisher Scientific (Rockfield, IL, USA). The H2AX (ab11175) primary antibody was purchased from Abcam (Cambridge, MA, USA). The NFkB (8008) primary antibody was from Santa Cruz Biotechnologies (Dallas, TX, USA). The γH2AX (NB100-384) primary antibody was from Novus Biologicals (Littleton, CO, USA). Goat anti-mouse Alexa Fluor 568 (A-11004) and goat anti-rabbit IgG Alexa Fluor 568 (A-11011) secondary antibodies and TRP53 (2524S) were purchased from Cell Signaling Technology (Danvers, MA, USA).

### Animal exposure and tissue collection

Female KK.Cg-a/a (*n* = 8) mice, designated as hyperphagic lean (HPL) and KK.Cg-Ay/J (*n* = 10), designated as hyperphagic obese (HPO), were obtained from Jackson Laboratories (Bar Harbor, ME, USA) at 6 weeks of age. Mice were housed two to five per cage in a facility maintained at 25°C with a 12-h circadian rhythm. All animal procedures were approved by the Iowa State University Institutional Animal Care and Use Committee. Food (2014 Envigo Teklad Global 14% Protein Rodent Maintenance Diet) and water were available to the animals *ad libitum*. Weekly food intake was measured in each cage biweekly and averaged to food consumed per mouse per day. Body weights were also monitored biweekly. The 7-day DMBA dosing period began at ∼9 weeks of age, ensuring acclimatization and a ~25% weight difference between the HPL and HPO mice. Mice received either corn oil as vehicle control (CT) or DMBA (1 mg/kg/day) via intraperitoneal injection. The dose of DMBA was based upon studies in which 14 d of dosing caused ovarian follicle loss [23] but the duration was reduced to 7 d to prevent complete loss of follicles in which to study molecular alterations that contribute to DMBA-induced ovotoxicity. Additionally, this identical exposure was used in the study from which the “immediate” ovary follicle numbers were derived [22] as comparison of the effect of a “recovery” period. Euthanasia occurred on day 2 of diestrus (8–22 d post-dosing) to ensure no variation in the estrous cycle hormonal milieu and to encompass the half-life of DMBA (~10 d) [34]. The delay in euthanasia post-dosing provided a recovery period from the immediate effects of exposure to DMBA. Total body, spleen, liver, ovary, and uterus weights were recorded, and ovaries were fixed in 4% paraformaldehyde overnight at 4^°^C and stored in 70% ethanol at 4^°^C thereafter.

### Estrous cycle monitoring

The estrous cycle was monitored daily by vaginal cytological assessment, starting at 7 days post-exposure and continued for an average of 8 days (range of 2–13 days) until day 2 of diestrus was reached by each animal and there was no treatment effect observed on the time to reach diestrus day 2. Briefly, the vagina was gently lavaged with saline solution (0.9%) and wet vaginal smears were observed directly under a light microscope. Stages of estrous were categorized as either proestrus, estrus, metestrus or diestrus phase based on the presence of three cell types, i.e. nucleated epithelial cells, cornified epithelial cells and leukocytes [35].

### Serum analysis

Blood was collected from mice via cardiac puncture at euthanasia and centrifuged at 10,000 rpm for 15 min at 4°C. The supernatant was collected, and the serum was analyzed by the Ligand Assay and Analysis Core Library at the University of Virginia. Progesterone was measured by radioimmunoassay (Calbiotech Mouse/Rat ELISA; n = 4 for HPL-CT and HPL-DMBA; n = 5 for HPO-CT and HPO-DMBA). The assay range was 0.15–40 ng/mL, and samples with a CV value >20 were disregarded. All samples were run in duplicates except one sample from the HPL-DMBA group due to low sample volume, and the average value was used. Samples were within the analytical range of the assay and the mean interassay CV was 5.4%.

### Follicle classification and enumeration

Paraffin-embedded ovaries (n = 4 for HPL-CT and HPL-DMBA; n = 5 for HPO-CT and HPO-DMBA) were sectioned at the Iowa State University Veterinary Medicine Histopathology Laboratory. Every sixth section, 5 μM thick, was mounted on a microscope slide, with two sections mounted per slide. Ovarian sections were stained with hematoxylin and eosin and slides were blinded before counting follicles to eliminate counter-bias. Healthy follicles that maintained structural integrity and contained an oocyte nucleus were counted on every 12th section using a Leica DM 500 microscope equipped with an ICC50W camera. Unhealthy follicles were distinguished by demonstration of pyknosis and intense eosinophilic staining. Follicular structures were classified as primordial, primary, secondary, pre-antral, and antral follicles as described [22] and the number of follicles in each ovarian section per ovary were totaled to calculate total follicle number. Corpora lutea, which are oocyte-devoid, were averaged across the sections for each ovary to avoid over-counting. The area of the CL was also measured. The follicle numbers were compared with ovaries collected immediately after cessation of DMBA exposure which were derived from [22]. This study used the same mouse strain, identical DMBA exposure paradigm and follicle counting was performed in the same way. Follicle numbers presented are those from every 12th section and a multiplication factor was not included.

### Protein localization and quantification

Ovarian tissue sections (one section per ovary; n = 4 for HPL-CT and HPL-DMBA; n = 5 for HPO-CT and HPO-DMBA) from each treatment group were stained by immunofluorescence to localize and quantify abundance of proteins of interest. Ovaries were sectioned in the same manner as for follicle classification. Tissues were warmed in a water bath (60°C, 30 min) followed by incubation in Citrasolv (3× for 5 min), rehydration in 100% (2 X for 3 min) and 70% ETOH (2 X for 3 min) and washing in PBS for 5 min. To restore epitope-antibody binding, tissue sections were heated in Tris base buffer (pH 9) in a water bath (95°C, 30 min) and allowed to cool (30 min). Sections were encircled with a hydrophobic PAP pen to retain solutions on the tissue section. Tissues were blocked with 5% goat serum in PBS (blocking buffer) for 1 h, then washed in PBS (3× for 5 min). Primary antibodies (dilutions provided in Table 1) were prepared in blocking buffer and applied to tissue sections overnight at 4°C. Slides were washed in PBS (3× for 5 min) and incubated in the appropriate secondary antibody for 1 h at room temperature in a darkened room. Slides were washed again in PBS (3× for 5 min), and coverslips were added using SlowFade Gold mounting media after blotting off any excess solution. Images were captured using the Leica DM6 B microscope fitted with a Lecia K5 camera at 501 nm for the cellular DNA stain, YOYO-1, and at 568 nm for the Alexa Fluor secondary antibodies. Images were acquired using Lecia Application Suite X software, and the intensity of the staining in specific follicle types was analyzed using ImageJ software. Primary-only, species-specific IgG antibody in place of primary antibody and secondary-only antibodies were performed as negative controls to confirm specificity of the quantified stain. Follicles without an antral cavity (primordial, primary, secondary) were classified as pre-antral, and the presence of a developing or developed antral cavity classified follicles as antral. The intensity of the stain for each protein was normalized by the area of the follicle. One section per ovary was stained and all sections for each protein were processed as a group. For statistical analysis, the staining intensity of follicles was averaged per ovary (n = 4 per treatment group).

**Table 1:** Dilutions of primary and secondary antibodies used for immunofluorescence staining

Primary/secondary antibody	Description	Dilution
H2AX	Ab11175	1:100
γH2AX	NB100-384	1:100
SOD1	PA5 27240	1:100
P53	CST 2524S	1:1,000
YOYO-1 iodide (491/509)	Thermofisher Y3601	1:5,000
Goat anti-Mouse IgG, Alexa Flour 568	Thermofisher A-11004	1:500
Goat anti-Rabbit IgG, Alexa Fluor 568	Thermofisher A-11011	1:500

### Statistical analysis

Statistical analyses were performed using GraphPad Prism v9.0 software. Unpaired t-test with Welch’s correction and one-way ANOVA with repeated measures were used to compare treatment groups. A two-way ANOVA function was used to analyze an interaction between obesity and DMBA exposure. Standard error of the mean (SEM) was used to report error bars. Statistical difference was reported at *P* < 0.05, whereas a tendency for a biologically meaningful statistical difference was considered at *P* < 0.10.

## Results

### Body weight increased in obese mice and recovery duration did not differ between treatment groups

**Figure 1:**
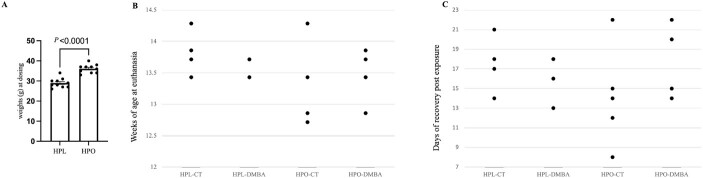
Body weight at onset of DMBA exposure, distribution of mouse age at euthanasia, and days spent in recovery post-DMBA exposure. Nine-week-old HPL or HPO mice were exposed to either corn oil (CT) or DMBA for 7 d. Euthanasia took place on day 2 of diestrus after a recovery period. (A) Body weight prior to DMBA exposure, (B) mouse age at euthanasia, and (C) days spent post-DMBA exposure. Note that some data points may overlap. n = 4 for HPL-CT and HPL-DMBA; n = 5 for HPO-CT and HPO-DMBA.

Lean and obese mice were dosed with DMBA for 7 days starting at ~9 weeks of age. At the beginning of dosing, the HPO group was ~25% heavier than the HPL group (Figure 1A; *P* < 0.0001). Post-exposure, animals had a recovery period (8–22 days) before ovary collection on the second day of diestrus. The individual age of mice at the time of euthanasia are presented in Figure 1B, and the average mouse age was HPL-CT: 13.82 ± 0.18 weeks, HPL-DMBA: 13.57 ± 0.08, HPO-CT: 13.20 ± 0.30, HPO-DMBA: 13.46 ± 0.17 weeks. Age of mouse at euthanasia did not differ (*P* > 0.05) between treatment groups. The distribution of the individual mouse recovery period in days is provided in Figure 1C, and the average length of recovery for each treatment was HPL-CT: 17.50 ± 0.18 days, HPL-DMBA:16.25 ± 1.18 days, HPO-CT: 14.20 ± 2.28 days, HPO-DMBA: 17.20 ± 1.59 days. The length of recovery time did not differ (*P* > 0.05) across treatments.

### Liver and spleen weight increased due to obesity, and uterine weight decreased due to DMBA exposure in obese mice

**Figure 2:**
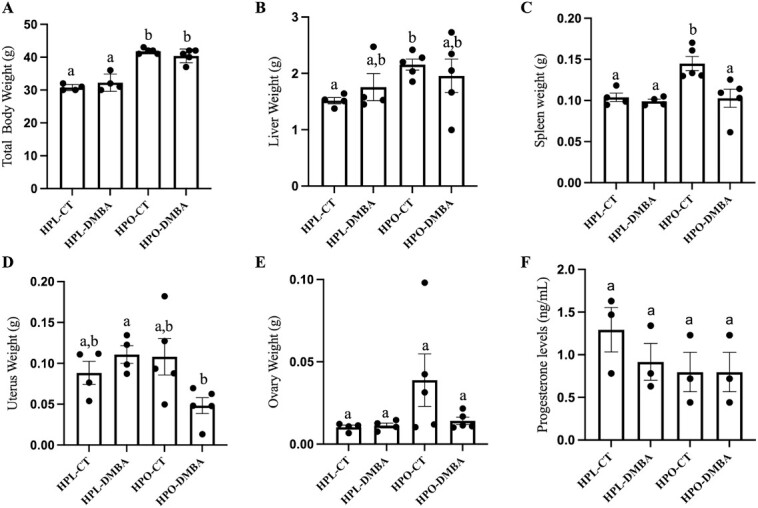
Body weight and circulating progesterone after DMBA recovery. Nine-week-old HPL or HPO mice were exposed to either corn oil (CT) or DMBA for 7 d. Weight of (A) body, (B) liver, (C) spleen, (D) uterus, and (E) ovary. (F) Serum progesterone level. Different letters indicate differences between treatments; *P* < 0.05; n = 4 for HPL-CT and HPL-DMBA; n = 5 for HPO-CT and HPO-DMBA.

Obese mice weighed more than their lean counterparts at the time of euthanasia (Figure 2A; *P* < 0.001). Obesity increased liver weight as compared with the lean control (Figure 2B; *P* < 0.05) but there was no effect of DMBA exposure (Figure 2B; *P* > 0.05). Obesity increased spleen weight (Figure 2C; *P* < 0.05), but this was not observed in either of the DMBA-treated mouse groups (*P* > 0.05). Exposure to DMBA reduced uterine weight in obese mice (Figure 2D; *P* < 0.05). Ovary weight was unaffected by obesity or DMBA exposure (Figure 2E; *P* > 0.05). Serum progesterone was also unchanged due to obesity or DMBA exposure (Figure 2F; *P* > 0.05).

### Obesity tended to reduce the number of primordial follicle and DMBA exposure tended to decrease the number of primary follicles in lean mice only

**Figure 3:**
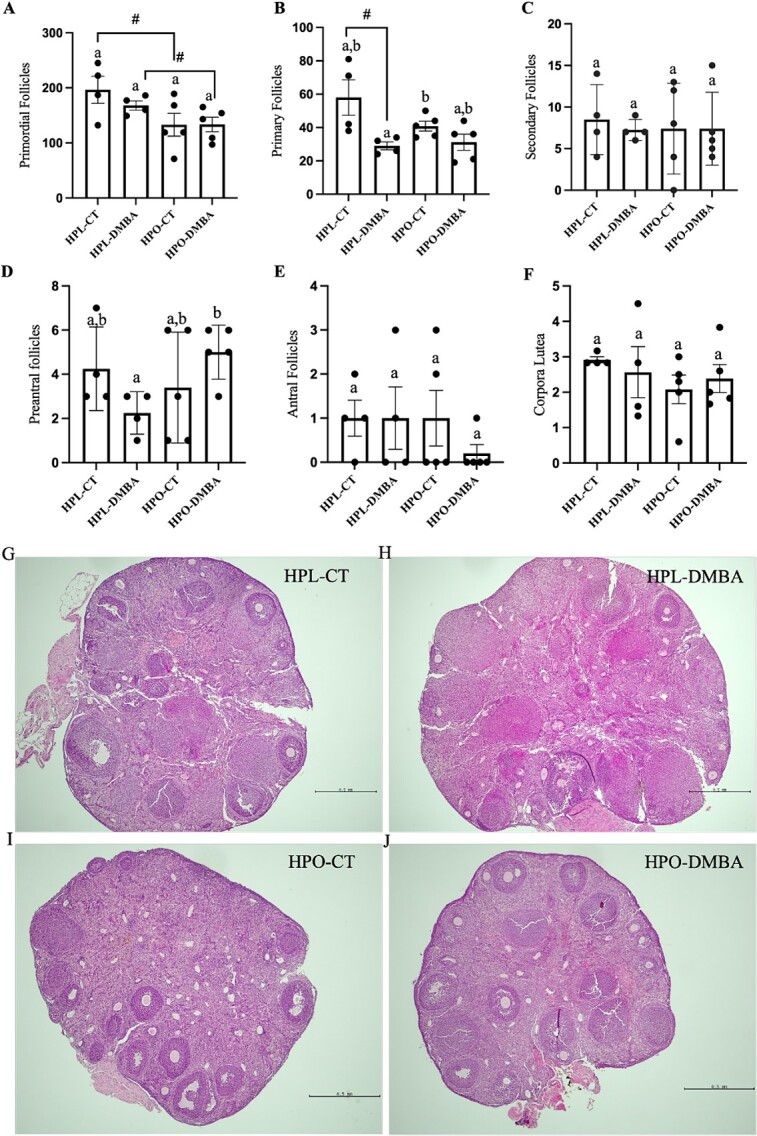
Follicle number in lean and obese mouse ovaries post-DMBA recovery. Nine-week-old HPL or HPO mice were exposed to either corn oil (CT) or DMBA for 7 d and allowed a recovery period. The number of (A) primordial (treatment effect *P* = 0.11; obesity effect *P* = 0.02; interaction effect *P* = 0.43), (B) primary (treatment effect *P* < 0.01; obesity effect *P* < 0.21); interaction effect *P* = 0.11), (C) secondary (treatment effect *P* = 0.76; obesity effect *P* = 0.81; interaction effect *P* = 0.76), (D) pre-antral (treatment effect *P* = 0.81; obesity effect *P* = 0.28; interaction effect *P* = 0.05), (E) antral follicles (treatment effect *P* = 0.45; obesity effect *P* = 0.45; interaction effect *P* = 0.45), and (F) corpora lutea (treatment effect *P* = 0.96; obesity effect *P* = 0.28; interaction effect *P* = 0.47) are presented. Different letters indicate differences between treatments; *P* < 0.05; # indicates *P* < 0.1; n = 4 for HPL-CT and HPL-DMBA; n = 5 for HPO-CT and HPO-DMBA. (G-J) are images of ovarian sections stained with hematoxylin and eosin from each treatment group with the scalebar = 0.5 mm.

There were no long-term effects of DMBA on healthy primordial follicle number (Figure 3A; *P* > 0.05) in lean mice, however obese mice tended to have fewer primordial follicles (Figure 3A; *P* < 0.1), regardless of DMBA exposure, compared with their lean control-treated counterparts. Exposure to DMBA tended to decrease primary follicle number in lean mice (Figure 3B; *P* < 0.1) but not in obese mice. Preantral follicle numbers were increased in DMBA-exposed obese compared with DMBA-exposed lean mice (Figure 3D; *P* < 0.05). The number of secondary follicles (Figure 3C; *P* > 0.05), antral follicles (Figure 3E; *P* > 0.05), and corpora lutea (Figure 3F; *P* > 0.05) were unaffected by obesity or DMBA exposure. When considering only body composition or DMBA exposure, an obesity effect was observed on primordial follicle number (*P* = 0.02), and a DMBA effect was observed on primary follicle number (*P* < 0.01).

### Primordial follicle loss persists post-DMBA exposure in obese mice and obesity alters pre-antral follicle dynamics

To examine sustained effects of DMBA exposure after the exposure has ceased, follicular number from lean and obese mice ovaries exposed to DMBA and collected immediately after dosing from a previously published study [22] designated “immediate” were compared with the follicle numbers in the ovaries in the current study – i.e. those who had a “recovery” period after cessation of DMBA exposure. The experimental design including animal models, doses, route of exposure, methods for counting follicles were same in the two comparative studies.

**Figure 4:**
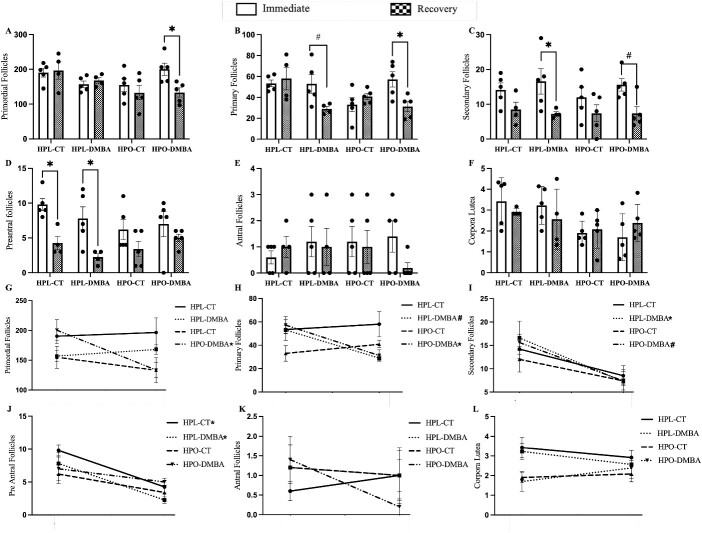
Temporal pattern of DMBA-induced follicle loss in lean and obese ovaries. Nine-week-old HPL or HPO mice were exposed to either corn oil (CT) or DMBA for 7 d with either “immediate” euthanasia (data published in reference 22) or after a “recovery” period. The number of (A, G) primordial, (B, H) primary, (C, I) secondary, (D, J) pre-antral, (E, K) and antral follicles, and (F, L) corpora lutea are presented for both time points. The trajectory of follicle loss in immediate to recovery ovaries is presented as line graphs (G–L). * = *P* < 0.05; # = *P* < 0.1; Immediate euthanized mice; n = 5 per group. Recovery euthanized mice; n = 4 for HPL-CT, HPL-DMBA and n = 5 for HPO-CT, HPO-DMBA.

After the recovery period, in obese mice exposed to DMBA, reduced numbers of primordial follicles were observed (Figure 4A, G; *P* < 0.05) but this was not noted in the lean mice which did not differ from the immediately collected ovary numbers. Loss of primary follicles continued in both lean (Figure 4B, H; *P* < 0.05) and obese (Figure 4B, H; *P* < 0.05) mice after the DMBA exposure had ceased. A similar pattern was observed for secondary follicle number where DMBA-exposed lean mice had reduced secondary follicle number (Figure 4C, I; *P* < 0.05), and DMBA-exposed obese mice tended to have reduced secondary follicle number (Figure 4C, I; *P* < 0.1) after the recovery period. Preantral follicle number were reduced in lean mice across the recovery timeframe without an influence of DMBA (Figure 4D, J; *P* < 0.05). Antral follicle number (Figure 4E, K; *P* > 0.05) and corpora lutea number (Figure 4F, L; *P* > 0.05) were unaffected across the recovery period.

### Both obesity and DMBA exposure reduced total ovarian H2AX

**Figure 5:**
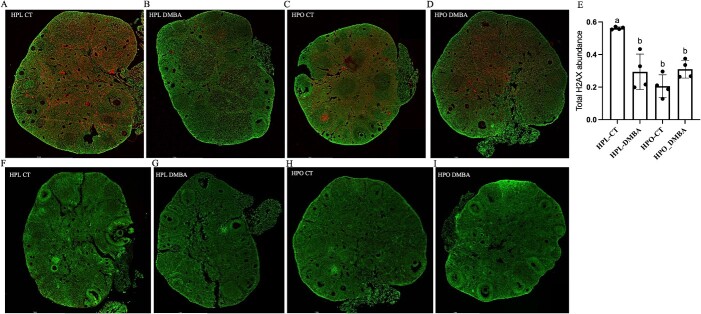
Ovarian H2AX and γH2AX staining in lean and obese ovaries post-DMBA exposure. Nine-week-old HPL or HPO mice were exposed to either corn oil (CT) or DMBA for 7 d and allowed a recovery period. Immunofluorescent staining to detect (A–D) H2AX (red) and (G–I) γH2AX (red) was performed in ovarian sections from all treatment groups. Total H2AX abundance was averaged per ovary in (E). The green stain represents cellular DNA. Images were captured at 20× magnification. Scale bar = 1 mm; n = 4 ovary sections from four individual mice per treatment.

To investigate if ovaries displayed markers of the DDR after the recovery from DMBA exposure, ovarian sections were immunostained for total histone H2A family member X (H2AX) and the phosphorylated active form, γH2AX. Total H2AX staining was visible in ovaries from mice of all treatment groups (Figure 5A–D), irrespective of cell type and follicle stage. The abundance of total H2AX was higher in lean control mice as compared with all other treatment groups (Figure 5E). Ovarian γH2AX was absent (Figure 5F-I) in control and DMBA exposed mice.

### A marker of oxidative stress is increased in corpora lutea of DMBA-exposed obese but not lean mice

**Figure 6:**
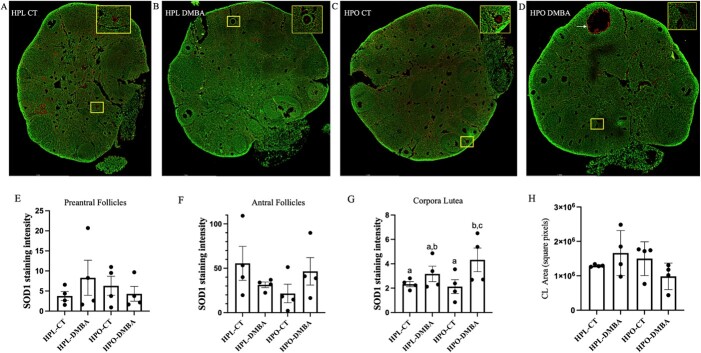
Ovarian SOD1 staining in lean and obese ovaries post-DMBA exposure. Nine-week-old HPL or HPO mice were exposed to either corn oil (CT) or DMBA for 7 d and allowed a recovery period. Immunofluorescent staining to detect SOD1 (red) was performed in ovaries from (A) HPL-CT, (B) HPL-DMBA, (C) HPO-CT, and (D) HPO-DMBA mice. The green stain represents cellular DNA. Images were captured at 20× magnification. Scale bar = 1 mm. The intensity of the stain was averaged per ovary in (E) pre-antral follicles, (F) antral follicles, and (G) corpora lutea. (H) The area of corpora lutea used for SOD1 abundance measurement was compared. n = 4 ovary sections from four individual mice per treatment. Different letters indicate differences between treatments, *P* < 0.05.

To investigate oxidative stress was present due to DMBA exposure, ovarian sections were immunostained to detect superoxide dismutase 1 (SOD1) and staining was visible in mostly atretic follicles (indicated in yellow boxes; Figure 6A-D) but also in the oocytes of some morphologically healthy appearing follicles (Figure 6B), particularly in DMBA-exposed lean mice. Quantification of SOD1 staining in different follicle types revealed no alteration in the abundance of SOD1 in small preantral follicles (Figure 6E; *P* > 0.05) or in large antral follicles (Figure 6F; *P* > 0.05) across the different treatment groups. However, the abundance of SOD1 was increased in the corpora lutea (Figure 6G; *P* < 0.05) of DMBA-exposed obese mice compared with the obese control treated mice. There was no impact (*P* > 0.05) on CL area due to obesity or DMBA exposure (Figure 6H).

### Obesity reduces TRP53 protein abundance in follicles of DMBA-exposed mice

**Figure 7:**
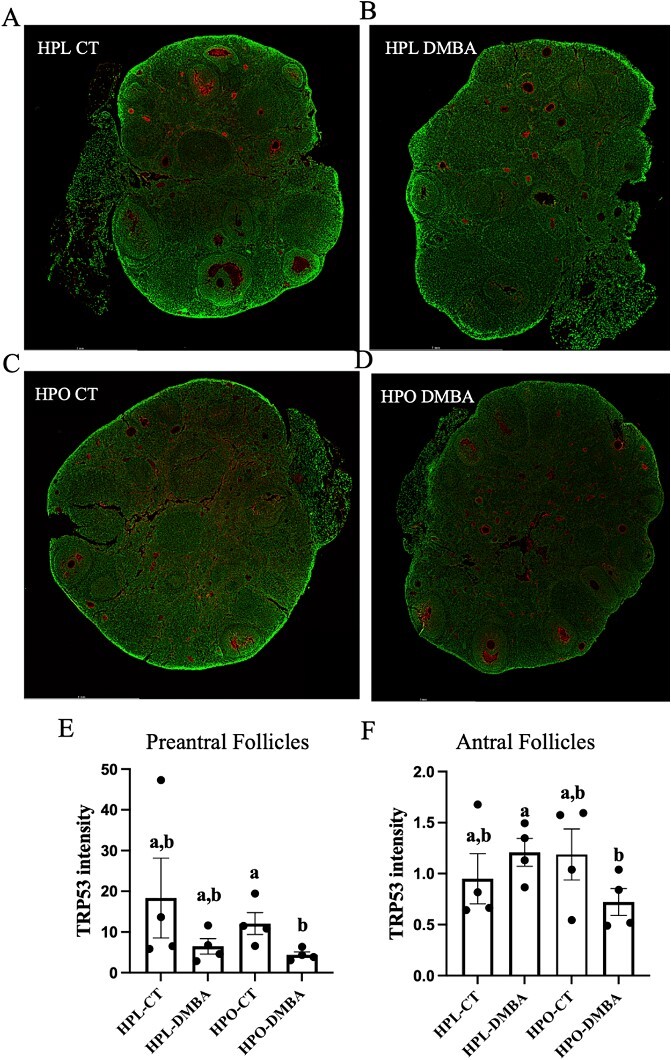
Ovarian TRP53 staining in lean and obese ovaries post-DMBA exposure. Nine-week-old HPL or HPO mice were exposed to either corn oil (CT) or DMBA for 7 d and allowed a recovery period. Immunofluorescent staining to detect TRP53 (red) was performed in ovaries from (A) HPL-CT, (B) HPL-DMBA, (C) HPO-CT, and (D) HPO-DMBA mice. The green stain represents cellular DNA. Images were captured at 20× magnification. Scale bar = 1 mm. The intensity of the stain was averaged per ovary in (E) pre-antral (F) and antral follicles; n = 4 ovary sections from four individual mice per treatment. Different letters indicate differences between treatments, *P* < 0.05.

To investigate the impact of DMBA on abundance of the tumor suppressor protein, cellular tumor antigen TRP53, lean and obese mouse ovarian sections were immunostained to detect TRP53. Localization of TRP53 was in the oocyte membrane of all follicle types and across all treatment groups (Figure 7A-D). The TRP53 staining was also visible in atretic follicles but appeared more diffused than in apparently healthy follicles. Quantification of healthy pre-antral follicle TRP53 staining revealed decreased TRP53 in DMBA-exposed obese mice as compared with obese control mice (Figure 7E; *P* < 0.05) and the same pattern of TRP53 staining was observed in healthy antral follicles with reduced TRP53 staining by DMBA exposure in obese compared with identically exposed lean mice (Figure 7F; *P* < 0.05).

## Discussion

Obesity is a complex and pervasive global health issue, characterized by excessive accumulation of body fat, leading to adverse health effects including, in females, hormonal imbalance, irregular menstrual cyclicity, ovulation issues, and infertility [36–38]. Obese mothers have a greater risk of miscarriage and birth defects in their offspring [39], potentially due to the impairments in oocyte quality [40]. In obese rodent models, greater ovarian damage has been observed due to environmental exposures [19, 21, 23, 25, 41]. However, the mechanisms of how obesity enhances ovotoxicity remain unclear.

A potent carcinogen and genotoxicant, DMBA causes the destruction of all ovarian follicle types [26]. Exposure to PAH chemicals is from incineration of organic matter, charring of foods and automobile exhaust [28]. In the ovary, DMBA is biotransformed into an ovotoxic metabolite that causes DNA adduct formation [29]. Double-stranded DNA breaks are highly cytotoxic and can be induced by DMBA since it is an alkylating agent [42]. Dose-dependent DNA damage caused by DMBA in extra-ovarian tissues has been previously reported [43] and extensive DMBA-induced follicle loss has been observed both in vivo [23, 44] and in vitro [45, 46] models.

In this study, lean and hyperphagia-induced obese mice were used to study the effects of both follicle loss continuation post-DMBA exposure, and to determine if difference exists between lean and obese mouse ovaries. Exposure to DMBA commenced when lean and obese mice were nine weeks old, to avoid the obesity-induced loss of primordial follicles observed in this model from 12 weeks of age onwards [20] with a targeted weight difference between the lean and the obese groups of ~25%. The half-life of DMBA is reported as 3.17 ± 1.1 d for phase I and 6.46 ± 1.3 d for phase 2 [34]. Thus, a “recovery period” ranging from 8 to 22 d incorporated both the half-lives of DMBA and ensured euthanasia at the same stage of the estrous cycle, i.e. day 2 of diestrus. Although there was variation across animals in the time to reach day 2 of diestrus, and thus variation in recovery period length, there was no treatment impact on the length of recovery time, thus the treatment comparisons are statistically valid.

The main phenotypic effects observed were due to obesity and not DMBA exposure. Excessive fat accumulation was visible in the abdomen of the mice in the obese group, and heavier livers were also recorded. Excessive fat accumulation in the liver is debilitating since it can impair liver function, increase the risk of liver disease, and trigger inflammation [47]. An increase in liver weight is a conserved phenomenon observed in prior studies involving this obese mouse strain [21, 22, 41] and while not surprising, recapitulated previous observations. Since obesity is a risk factor for non-alcoholic fatty liver disease [48], future studies to investigate the impact of fatty liver during ovotoxicant exposures are warranted. Spleen weights were also elevated in obese control mice compared with lean control and splenomegaly is also associated with liver disease [49]. Variation in the impacts of obesity on spleen weight is reported with heavier [19] and unaltered spleen weight being noted with this strain of mouse [22, 41]. A decrease in uterine weight was observed in the DMBA-exposed obese mice, but not the lean mice so whether this is attributable to endocrine disruption was not clear since no changes to P_4_ levels were observed. The obese control mice spent less time in the estrus phase of their estrous cycle, which could translate to reduced fertility [50]. This is in congruence with pervious work with the agouti lethal yellow mice wherein obesity affected estrous cyclicity by increasing the length of the diestrus phase and concomitantly decreasing the length of the estrus phase [20]. Leptin is an adipocyte-derived satiety factor, and the agouti lethal yellow mice have hyperleptinemia, eventually developing leptin resistance, and leptin resistance has been previously linked with early reproductive senescence [51]. Obese mice exposed to DMBA, spent a shorter time in metestrus and diestrus relative to the vehicle control-treated obese mice, although this is likely attributable to the increased length of time spent by the vehicle-control treated obese mice at this stage, which is associated with ovarian failure. Thus, obesity altered organ weights and there were impacts on ovarian function both in obese and in DMBA-exposed mice.

In humans, studies on the association between obesity and the ovarian reserve have been contradictory. Some show no correlation between obesity and ovarian reserve but have had limited sample size in the study design [52–54] whereas a study with a much higher sample size determined a negative correlation between AMH levels and BMI [55]. A negative correlation between obesity and AMH has also been reported in which lower AMH levels were attributed to other physiological processes rather than specifically the ovarian reserve [56]. Nonetheless, diminished ovarian reserve in women who smoke cigarettes (a DMBA source) has been reported [57–59] and both obesity and DMBA exposures combined reduced ovarian follicle numbers [20, 23]. Effects of PAH exposures on the ovarian reserve in offspring have also been reported [60], thus, the number of primordial follicles which comprise the ovarian reserve was a focus of this study and we had the opportunity to compare ovarian follicle number effects of DMBA exposure after a recovery period, with those collected immediately post-exposure. After the recovery period, the main follicle composition observation was that follicle numbers were impacted minimally in the DMBA-exposed or the obese groups with the exception of pre-antral follicles which differed between lean and obese DMBA-exposed mice being increased in the latter group. To capture if DMBA instigated follicle loss that continued post-exposure, follicle numbers at each stage (“recovery”) were compared with those from a previous separate study (“immediate”) that employed the same experimental design, except that mice were euthanized immediately post-cessation of DMBA exposure [22]. After the recovery period, numbers of primordial follicles, which comprise the ovarian reserve, were lower in obese but not lean mice due to the DMBA exposure. This suggests that, in obese mice, some checkpoint has been altered that permits loss of primordial follicles to continue post-exposure and is a concern for the ovarian reserve. This observation could indicate that in the obese mice, DMBA is activating primordial follicles to enter the pool of growing follicles as a mode of toxicity. Accelerated primordial follicle activation as a mode of ovotoxicity has been reported previously [45] and while not considered a mode of action of DMBA in the lean female, these findings suggest that metabolic alterations during obesity induce primordial follicle activation as a consequence of DMBA exposure.

In both lean and obese mice, loss of primary follicles due to DMBA exposure continued across the recovery period, albeit a statistical trend in the lean mice. This pattern of follicle loss suggests that primary follicles that were already activated to die did not have the capacity to overcome the DMBA-induced damage instigated. This same pattern was true in secondary follicles, with continued loss of secondary follicles in both lean and obese mice, albeit this time the statistical trend was in the obese compared with the lean mice, however the pattern holds and aligns with the primary follicle findings that once death pathways were induced, they could not be overcome. For preantral follicles, a differential effect was noted between lean and obese mice, and regardless of DMBA exposure, preantral follicles were reduced in lean mice across the recovery period but this was absent in obese mice. In the lean mice, this is likely reflective of normal ovarian physiological processes involved in the recruitment and maturation of follicles toward ovulation and is likely a consequence of the mice being post-ovulation. In the obese mice, this observation was absent, suggesting perturbations to folliculogenesis due to obesity. Post preantral follicle stages were unaltered by obesity or DMBA exposure. The observation of the primordial follicle reduction coupled with the reduction in primary and secondary follicles suggests that primordial follicles are being hyperactivated into the growing follicle pool by DMBA exposure in the obese but not the lean mice.

To understand molecular alterations during the recovery period from DMBA-induced ovotoxicity, several molecular biomarkers were assessed via immunostaining to assess follicular location and quantification. Genotoxicity induced by DMBA is attributed to its alkylating properties leading to double-stranded breaks (DSBs) [42]. Despite total H2AX staining being evident in all treatment group ovaries with reduced H2AX in lean DMBA exposed and both obese groups, the gold standard marker for DSBs, γH2AX [61, 62], was absent, indicating that the immediate DDR was not ongoing. Previously, increased γH2AX was observed in older (18-week-old) mice exposed to DMBA for 14 days [33] and in the ovaries collected immediately after exposure that were used for the follicle count comparisons [22], providing evidence that the DDR had been activated but had ceased after a recovery period from DMBA exposure. Reduced total H2AX due to DMBA exposure in the lean and by obesity regardless of DMBA exposure raise further concern about the capacity of the obese ovary for DNA repair.

Whether the DMBA-induced continuation of follicle loss was attributable to oxidative stress was queried and SOD1 was used as an oxidative stress biomarker [63–65]. Immunolocalization of SOD1 revealed the presence of oxidative stress throughout the ovary in all treatment groups and with no apparent pattern of distribution. SOD1 can also act as a nuclear transcription factor [66] and staining was localized to the nucleus in healthy follicles but appeared more diffuse in atretic follicles. The staining intensity quantification demonstrated that SOD1 was increased in the corpora lutea of DMBA-exposed obese mice which was surprising since no effect of obesity or DMBA exposure on corpora lutea number or area were noted. However, since reactive oxygen species are also produced within the ovary during ovulation and luteinization of the regressing follicle and are critical determinants of corpora lutea lifespan [67], it could be that DMBA exposure in obese mice is altering an aspect of ovarian biology not investigated herein and perhaps is attributable to accelerated ovarian aging [68, 69].

Signaling by the transcription factor TRP53 regulates genes involved in cell cycle arrest, apoptosis and senescence [70] and is being explored as an emerging anti-cancer therapeutic approach [71–73]. Exposure to DMBA has also been demonstrated to reduce *Trp53* mRNA abundance prior to follicle loss in DMBA-exposed cultured ovaries [74] and has been associated during DMBA-induced ovarian carcinogenesis [75]. Additionally, TRP53 is increased during follicle loss induced by bisphenol A in cultured ovaries [76]. Although TRP53 functions as a transcription factor, localization of TRP53 was primarily observed in the oocyte oolemma. In antral follicles, TRP53 protein is reported in granulosa cells [77], but TRP53 has not been previously identified as an abundant protein in the oolemma [78]. In pre-antral and antral follicles of DMBA-exposed obese mice, TRP53 protein was reduced, potentially indicating that the temporal pattern of the response to DMBA-induced DNA damage has been altered or that it is impaired. Since TRP53 has multiple reports of importance for maintaining ovarian health [79, 80], including regulation of the primordial follicle reserve [81], this reduction could have local intra-ovarian consequences.

Taken together, the findings reported herein suggest that DMBA-induced loss of primordial follicles, once initiated, is sustained in the obese mouse ovary, potentially through a compromised DDR or activation of primordial follicles into the growing pool. Additionally, DMBA-induced loss of primary and secondary follicles continues after the exposure has ceased indicating that once initiated, the death pathway is irreversible. Increased SOD1 in the corpora lutea and decreased TRP53 abundance in pre-antral and antral follicles of the DMBA exposed obese ovary suggest induction of oxidative stress and a compromised DDR due to obesity. Reduced total H2AX, a DNA damage sensor, is also reduced by obesity further indicating that DNA repair could be impaired by obesity. These findings add to understanding the dynamics of follicle loss caused by metabolic perturbation and ovotoxicant exposure.

##  

**Conflict of Interest:** The authors have declared that no conflict of interest exists.

## Author Contributions

AFK contributed to experimental conception and design. KT and HEW performed animal study and tissue collection. KCK provided intellectual and technical assistance with microscopy. JKR performed the experiments and data analysis. JKR drafted the manuscript. AFK reviewed, edited and approved the final manuscript.

## Data availability

Data available on request.
